# Incidence of miscarriages in women with children with 47,XXY, 48,XXXY, or 49,XXXXY

**DOI:** 10.3389/fendo.2025.1688843

**Published:** 2025-11-26

**Authors:** Elizabeth Moser, Margaret Olaya, Andrea Gropman, Teresa Sadeghin, Carole Samango-Sprouse

**Affiliations:** 1 Department of Research, The Focus Foundation, Davidsonville, MD, United States; 2 Department of Pediatric Medicine, St. Jude Children’s Research Hospital, Memphis, TN, United States; 3 Department of Pediatrics, George Washington University School of Medicine and Health Sciences, Washington DC, United States; 4 Department of Human and Molecular Genetics, Florida International University, Miami, FL, United States

**Keywords:** miscarriage, sex chromosome aneuploidy, Klinefelter syndrome, 47,XXY, 48,XXXY, 49,XXXXY

## Abstract

**Purpose:**

The management and the causes of miscarriages present challenges for the obstetrical community as well as for families. In families who have a child with an X and Y chromosomal disorder, the duress of pregnancy loss may be significantly exacerbated. A greater understanding of these mechanisms would inform medical providers and families of children with 47,XXY, or Klinefelter syndrome (KS), and variant disorders (48,XXXY and 49,XXXXY). This study investigates miscarriage incidences across a cohort of mothers of male offspring born with these disorders.

**Methods:**

Pregnancy history of mothers of male offspring with 47,XXY (KS) or variant disorders was collected. Statistical analyses were performed to determine if these mothers experienced higher incidences of miscarriage than the general population.

**Results:**

Mothers reporting miscarriage in the 47,XXY (*p* = 0.03, *d* = 0.31) and 48,XXXY (*p* = 0.02, *d* = 1.95) groups were significantly older at the time of birth than those who did not report miscarriage. When compared to known statistics of miscarriage in the general population, there was a significant increase in the miscarriages in the 47,XXY (*p* = 0.04, *h* = 0.13), 49,XXXXY (*p* = 0.02, *h* = 0.23), and combined groups (*p* < 0.01, *h* = 0.15).

**Conclusion:**

Mothers of children with 47,XXY (KS) and variant disorders are at increased risk of miscarriage compared to the general population based on the findings of this study.

## Introduction

Miscarriage and repeated pregnancy loss (RPL) present challenges for the obstetrical community and emotional repercussions with associated negative quality of life effects for families (1). For families who have a child with sex chromosomal aneuploidies (SCAs), the stress of pregnancy loss may be significantly exacerbated; therefore, a greater understanding of these mechanisms could be helpful to medical providers and families of children with 47,XXY, 48,XXXY, and 49,XXXXY. Approximately 15% of clinically recognized pregnancies end in a sporadic and spontaneous miscarriage, with the leading cause of these losses (approximately 60%) arising from aneuploid errors (2).

Due to fertility decline, the risk of non-disjunction is increased in women of advanced maternal age (35 years and older). Non-disjunction errors result in an increased risk of genetic abnormalities during pregnancy as well as increased risk for subsequent sporadic aneuploid miscarriages (2, 3). By 40 years old, sporadic aneuploid miscarriage rates reach 50% (2). However, these miscarriages are assumed to occur randomly; therefore, history of the first aneuploid miscarriage does not increase the risk of subsequent aneuploid miscarriages (4).

Two or more failed clinical pregnancies are considered RPL and affect 1%–2% of women of childbearing age (2). The pathogenesis of RPL is not fully understood; however, uterine abnormalities, hormonal disorders, chromosomal errors, and lifestyle factors are possible influences (5). Advanced maternal age may also increase the risk of RPL (6). The risk of RPL after two miscarriages rises from 24% at 25–29 years old to 44% at 40–44 years old (6). However, aneuploidy is not always associated with RPL, as sporadic aneuploid miscarriages occur randomly and are thought to affect mothers with and without history of RPL (7). Additionally, an aneuploid miscarriage is linked with better prognoses for subsequent pregnancies than euploid miscarriage (7).

47,XXY, 48,XXXY, and 49,XXXXY are SCAs that are compatible with life and result most often from non-disjunction during meiotic division of gametogenesis or, less often, from mitotic non-disjunction in zygotes (8). Studies have shown that, unlike other trisomy disorders, SCAs have a nearly equal chance of resulting from a meiotic non-disjunction event in the father as in the mother. Moreover, advanced maternal age has also been found to have an effect on the incidence of postzygotic mitotic non-disjunction resulting in Klinefelter syndrome (KS) (9). The incidence rate for each variant is estimated to be approximately 1:500 to 660 live births for male offspring with 47,XXY, 1:50,000 live births for male offspring with 48,XXXY, and 1:85,000 to 100,000 live births for male offspring with 49,XXXXY (10, 11).

There is wide variability in phenotypic presentations of male offspring with these disorders, with each additional X chromosome potentially increasing the severity of the known deficiencies (10). These individuals have demonstrated hypergonadotropic hypogonadism, truncal hypotonia, language learning difficulties, behavioral problems, and IQs ranging from above average to moderately impaired, with a dosage effect of each additional X chromosome (10). There is no evidence to suggest that recurrence rates in families with children with SCAs are higher than those of the general population; however, a recent study indicates that SCAs might be more likely to occur in pregnancies of younger women and may be associated with previous pregnancy loss (12).

The association between SCA conditions, miscarriage, and recurrent pregnancy loss has been underinvestigated at this time. The introduction of non-invasive prenatal testing (NIPT) has increased the detection of these disorders, and providing the medical community and families of children with SCAs with critical information regarding miscarriage risk would promote a more informed approach to obstetrical care in these cases (13). To our knowledge, there have not been any comprehensive studies documenting incidences of miscarriage in women who deliver children with SCA. The present study aims to investigate miscarriage incidences across a worldwide cohort of mothers of male offspring born with 47,XXY and variant disorders (48,XXXY and 49,XXXXY).

## Methods

This is a retrospective study of 497 male offspring with a karyotype of 47,XXY, 30 male offspring with 48,XXXY, and 130 male offspring with 49,XXXXY. At the time of enrollment, detailed health histories were obtained from the families. The inclusion criteria for this investigation included a confirmed 47,XXY, 48,XXXY, or 49,XXXXY diagnosis via chromosomal microarray analysis and/or karyotype and a complete pregnancy history. Each mother’s pregnancy history included the number of pregnancies, live births, miscarriages, and age at the time of birth to a child with the disorder and was treated as an individual data point. Mothers who did not supply a complete pregnancy history or who had male offspring with these disorders who were found to have a mosaic karyotype, have copy-number variants, and/or any other co-existing genetic disorders were excluded from the study to reduce confounding factors and provide greater fidelity of data. The incidence of miscarriages among the population of mothers of children with all three disorders (KSc) was compared to the known incidence of miscarriage in the general population (15%) (2). Miscarriage data are not regularly, systematically collected in the USA, so this incidence rate served as the control group for our study.

This study was reviewed and approved by The Western Institutional Review Board, which also approved this study’s protocol (#20081226). Written informed consent to participate in this study was provided. Of the initial group of women who had children with these disorders, 231 mothers of male offspring with 47,XXY, 12 mothers of male offspring with 48,XXXY, and 88 mothers of male offspring with 49,XXXXY qualified for the investigation.

Statistical analyses were completed in *jamovi* software version 2.6 (14). Descriptive statistics were performed to identify the percentage of clinical miscarriages and incidences of RPL within each cohort and across all groups (KSc). Independent sample *t*-tests were used to determine whether there was a significant difference between the average maternal age of mothers with 47,XXY (KS) who reported a history of miscarriage and those who did not report miscarriage within each group. Independent sample *t*-tests were also used to explore whether there was a significant difference in the average maternal age of mothers who reported a history of one miscarriage and those reported RPL within each group. Chi-square tests of independence were used to examine whether there was an association between maternal age at the time of birth and reported miscarriage within each of the three groups. One-sample tests of proportions were used to evaluate whether there was a significant difference in the proportions of mothers in the KSc group who reported miscarriage versus the proportion of mothers in the general population who reported miscarriage. All *p*-values were compared to alpha = 0.05.

## Results

### Mothers of male offspring with 47,XXY

#### Descriptives

Measures of central tendency and dispersion were computed to summarize age data. The mean age of the mothers at the time of birth to male offspring with 47,XXY was 35.1 years (*n* = 231, *s* = 5.35). The age distribution approached normal, with a skewness of −0.30.

In the cohort of women who had a child with 47,XXY, 19.9% (*n* = 46) reported a history of miscarriages. Of those with a reported history of miscarriage, 63.0% (*n* = 29) of women reported having only one miscarriage, and 48.3% (*n* = 14) were of advanced maternal age (AMA). Of the original sample, 7.35% (*n* = 17) experienced RPL. Of those reporting RPL, 88.2% (*n* = 15) were of AMA.

#### Independent sample *t*-tests

The mean age of the mothers with male offspring with 47,XXY who reported a history of miscarriage ( $\bar{x}$ = 36.46) was significantly higher than those in the cohort who did not report miscarriage ( = 34.82), *t*(229) = 1.87, *p* = 0.03, *d* = 0.31. The mean age of the mothers in this group who reported a history of one miscarriage ( = 38.3) was not significantly different than those in this cohort who reported RPL ( $\bar{x}$ = 39.8), *t*(44) = 0.983, *p* = 0.33, *d* = 0.30 (**Table 1**).

**Table 1:** Results comparing miscarriage proportions to the incidence in the general population (15%) using one-sample tests of proportion.

Population Subset	*n*	Statistic	Observed proportion (%)	*p*	Cohen’s *h*
Mothers of male offspring with 47,XXY	231	2.09	19.9	0.04*	0.13
*AMA removed*	99	0.61	17.2	0.54	0.06
Mothers of male offspring with 48,XXXY	11	0.30	18.18	0.77	0.09
Mothers of male offspring with 49,XXXXY	88	2.33	23.86	0.02*	0.23
*AMA removed*	70	2.18	24.29	0.03*	0.24

AMA, advanced maternal age; KSc, Klinefelter syndrome and variants.

*Indicates statistical significance compared to *α* = 0.05.

#### Chi-square tests of independence and single-sample proportion testing

**Figure 1:**
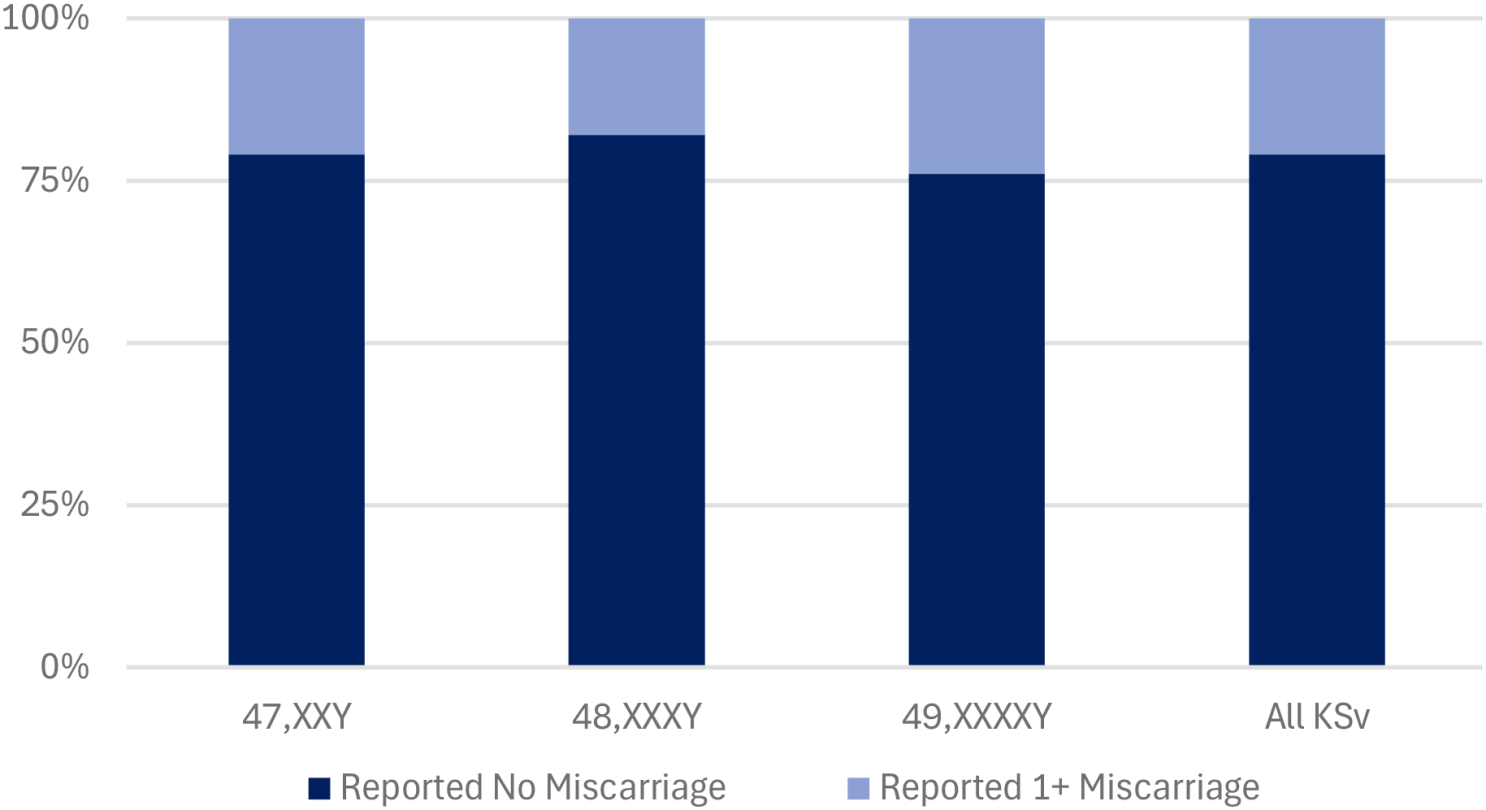
Reported miscarriage proportions of mothers of male offspring with KSc. Mothers are organized by KSc. The percentage of mothers who did not report miscarriage is represented in dark blue. The percentage of mothers who reported one or more miscarriages is represented in light blue.

No significant association between maternal age and miscarriage history was found in this cohort, *X*^2^ (1, *N* = 231) = 0.82, *p* = 0.37, *ϕ* = 0.06. Moreover, a single-sample proportion test was used to compare the proportion of mothers of male offspring with 47,XXY who reported a history of miscarriage to the proportion of women in the general population who report a history of miscarriage. There was a significant difference in the proportions of miscarriage in 47,XXY when compared to the general population, 19.9%, *z*(231) = 2.09, *p* = 0.04, *h* = 0.13. When mothers of AMA in this cohort were removed from the sample, there was no significant difference observed between the mothers of male offspring with 47,XXY to the general population, 17.2%, *z*(99) = 0.61, *p* = 0.54, *h* = 0.06 (**Table 1**; **Figure 1**).

### Mothers of male offspring with 48,XXXY

#### Descriptives

Measures of central tendency and dispersion were computed to summarize age data. The mean age of mothers with male offspring with 48,XXXY was 30.7 years (*n* = 11, *s* = 7.55). Two modes exist in the dataset, specifically 20 and 36 years. The age distribution approached normal, with a skewness of 0.66.

Two women (18.2%) in this group had a history of miscarriage, with one mother reporting a single miscarriage, the other reporting RPL, and both were of AMA. All miscarriages were reported in AMA women (**Table 2**). The mean age of the two mothers who had male offspring with 48,XXXY and miscarriages ( $\bar{x}$ = 40.5) was significantly higher than those in the cohort who did not miscarry ( = 28.6).

**Table 2:** Reported miscarriage frequencies by maternal age at the time of birth to a child with KSc.

Population Subset	Reporting one miscarriage		Reporting more than one miscarriage		
	nAMA	AMA	nAMA	AMA	Sum (%)
Mothers of male offspring with 47,XXY (*n* = 231)	15	14	15	2	46 (19.9)
Mothers of male offspring with 48,XXXY (*n* = 11)	0	1	0	1	2 (18.2)
Mothers of male offspring with 49,XXXXY (*n* = 88)	13	4	4	0	21 (23.8)
All mothers of male offspring with KSc (*n* = 330)	28	19	16	6	69 (20.9)

nAMA, not of advanced maternal age; AMA, of advanced maternal age.

### Mothers of male offspring with 49,XXXXY

#### Descriptives

Measures of central tendency and dispersion were computed to summarize age data. The mean age of the mothers who had a male offspring with 49,XXXXY was 30.6 years (*n* = 88, *s* = 4.77). The age distribution approached normal, with a skewness of −0.14. A total of 23.8% (*n* = 21) of women reported a history of miscarriage, and only 19% (*n* = 4) were of AMA. Moreover, 80.9% (*n* = 17) of the cohort reported only one miscarriage, and 4.5% (*n* = 4) reported RPL. In mothers who had one miscarriage, 23.5% (*n* = 4) were of AMA, and in those who had RPL, none were AMA.

#### Independent sample *t*-tests

The mean age of those mothers who had a history of miscarriage ( $\bar{x}$ = 31.5) was not significantly higher than those who did not ( $\bar{x}$ = 30.3), *t*(86) = −1.00, *p* = 0.16, *d* = 0.25. The average age of those who had one miscarriage ( = 31.6) was not significantly different than those who had RPL ( $\bar{x}$ = 31.0), *t*(19) = −0.3, *p* = 0.77, *d* = 0.17 (**Table 3**).

**Table 3:** Comparing the average age of mothers at the time of birth to KSc by reported RPL using one-tailed *t*-tests.

Population Subset	Number of miscarriages	*n*	*M*	SD	Statistic	*df*	*p*	Cohen’s *d*
Mothers of male offspring with 47,XXY	>1	17	39.8	5.01	0.98	44	0.33	0.30
	1	29	38.3	4.75				
Mothers of male offspring with 49,XXXXY	>1	4	31.0	4.24	−0.30	19	0.77	0.17
	1	17	31.6	3.79				
All mothers of male offspring with KSc	>1	22	38.3	5.85	3.65	67	<0.001*	0.94
	1	47	33.6	4.54				

*Indicates statistical significance compared to *α* = 0.05. RPL, repeated pregnancy loss.

#### Chi-square tests of independence and single-sample proportion testing

No significant association between maternal age and miscarriage history was identified in this cohort, *X*^2^ (1, *N* = 88) = 0.03, *p* = 0.86, *ϕ* = 0.02. There was a significant difference in the proportions between the mothers of boys with 49,XXXXY and the general population, 23.9%, *z*(88) = 2.33, *p* = 0.02, *h* = 0.23. When mothers of AMA were removed from the sample, a significant difference remained between the mothers of boys with 49,XXXXY and the general population, 24.29%, *z*(70) = 2.18, *p* = 0.03, *h* = 0.24 (**Table 1**, **Figure 1**).

### All mothers of male offspring with KSc

#### Descriptives

The three groups were combined to see if the larger cohort revealed any additional insights into the risk of miscarriage. Measures of central tendency and dispersion were computed to summarize age data. The mean age of the mothers with male offspring with KSc was 33.8 years (*n* = 330, *s* = 5.66). The age distribution approached normal, with a skewness of −0.21.

In this cohort of mothers, 20.9% (*n* = 69) reported a history of miscarriage with 68.1% (*n* = 47) reporting one miscarriage (**Table 2**). Of those who reported only one miscarriage (51%, *n* = 35), 40.4% (*n* = 19) were of AMA at the time of birth. Seven percent (*n* = 22) of the sample reported RPL and 72.7% (*n* = 16) of those reporting RPL were AMA.

#### Independent sample *t*-tests

The mean age of the mothers who had a male offspring with KSc who reported a history of miscarriage ( $\bar{x}$ = 35.1) was significantly higher than those who did not report miscarriage ( $\bar{x}$ = 33.4), *t*(328) = 2.13, *p* = 0.02, *d* = 0.29. The average age of the mothers who reported RPL ( $\bar{x}$ = 38.3) was significantly higher than those who reported a history of one miscarriage ( $\bar{x}$ = 33.6), *t*(67) = 3.65, *p* < 0.001, *d* = 0.94 (**Table 3**).

#### Chi-square tests of independence and single-sample proportion testing

No significant association between each of the KSc and reporting miscarriage history was found across the entire cohort, *X*^2^ (2, *N* = 330) = 0.03, *p* = 0.72. Furthermore, no significant association between maternal age and reporting a positive miscarriage history was found when analyzing the entire cohort, *X*^2^ (1, *N* = 330) = 0.58, *p* = 0.45, *ϕ* = 0.04.

A single-sample proportion test was used to compare the proportion of mothers of male offspring with KSc who reported a history of miscarriage to the known proportion of women in the general population who report miscarriage. A significant difference was found between the two groups, 20.9%, *z*(330) = 3.01, *p* < 0.01, *h* = 0.15. When mothers of AMA were removed, there was no significance found between the two groups, 19.3%, *z*(176) = 1.60, *p* = 0.11. *h* = 0.11 (**Table 1**; **Figure 1**).

## Discussion

This study reports on a large cohort of families of children with 47,XXY, 48,XXXY, and 49,XXXXY and incidences of miscarriages and recurrent pregnancy loss. The occurrence of miscarriage was significantly higher for all mothers of male offspring with KSc compared to the general population. When mothers were trifurcated into groups according to the number of additive Xs, mothers of male offspring with 47,XXY and 49,XXXXY experienced significantly higher proportions of miscarriage compared to the general population: 19.9% and 23.8%, respectively. These findings document that mothers of children with SCA disorders are at a higher risk of miscarriage, probably due to non-disjunction.

Mothers of male offspring with 48,XXXY may also be at increased risk; however, our study’s cohort was small, likely due to poor ascertainment of this disorder. These boys have a few dysmorphic features or congenital abnormalities associated with their developmental delay, so typically, they may not be referred for a genetic evaluation. Additionally, 48,XXXY is not specifically identified through NIPT, leading to decreased detection prenatally. Therefore, the risk of miscarriage in mothers of children with 48,XXXY warrants further investigation in the future as ascertainment improves.

Mothers of children with 47,XXY and 48,XXXY who miscarried were significantly older than their counterparts who did not. Our findings describe that mothers of male offspring with 47,XXY and 48,XXXY are at increasing vulnerability of miscarriage as their age increases. In contrast, 81% of mothers in the 49,XXXXY group who had miscarriages were not of AMA. However, this cohort was also small, likely due to the rarity of the disorder. Furthermore, there was no significant difference in age found between mothers who reported miscarriages and those who did not. These findings support the idea that mothers of children with 49,XXXXY are predisposed to non-disjunction and have a higher risk of miscarriage at younger ages.

Mothers of children with KSc reported higher RPL; however, after isolating the mothers who were of AMA, occurrences approached those of the general population for mothers of male offspring with 47,XXY. In contrast, mothers of male offspring with 49,XXXXY reported higher RPL occurrences (4.5%) and were not of advanced maternal age. This finding further demonstrates that mothers of male offspring with 49,XXXXY may be predisposed to non-disjunction and have a higher risk of RPL regardless of increasing age.

The availability of data related to miscarriage incidence in the general population of the United States is limited, including incidence broken down by age, making it difficult to assess the relationship between miscarriage incidence and maternal age. However, in 2019, a comprehensive Norwegian study collated miscarriage data and reported incidence rates by age (15). The reported incidence rates that correspond to the average age of the mothers in each of our cohorts are not significantly different from the incidence rates of the general population (15%) used in this study for comparison.

Overall, there is an increased risk of miscarriage and RPL in mothers of male offspring with KSc, with implications of potential risk variability by disorder, as well as maternal age. Specifically, mothers of children with 49,XXXXY are at increased risk for miscarriage throughout the life cycle of reproduction, in contrast to mothers of 47,XXY whose risks increase with advancing age, similar to the general population. This investigation highlights the need for increased guidance and support for mothers who have a child with SCA in future pregnancies and the possible risk for miscarriage.

## Data Availability

The raw data supporting the conclusions of this article will be made available by the authors, without undue reservation.
